# Supplementary figures and images for: Detection of microplastics in Litopenaeus vannamei (Penaeidae) and Macrobrachium rosenbergii (Palaemonidae) in cultured pond
Source: PeerJ. 2022 Feb 8;10:e12916. doi: 10.7717/peerj.12916 (PMC8833223; doi:10.7717/peerj.12916)

## Slide 1
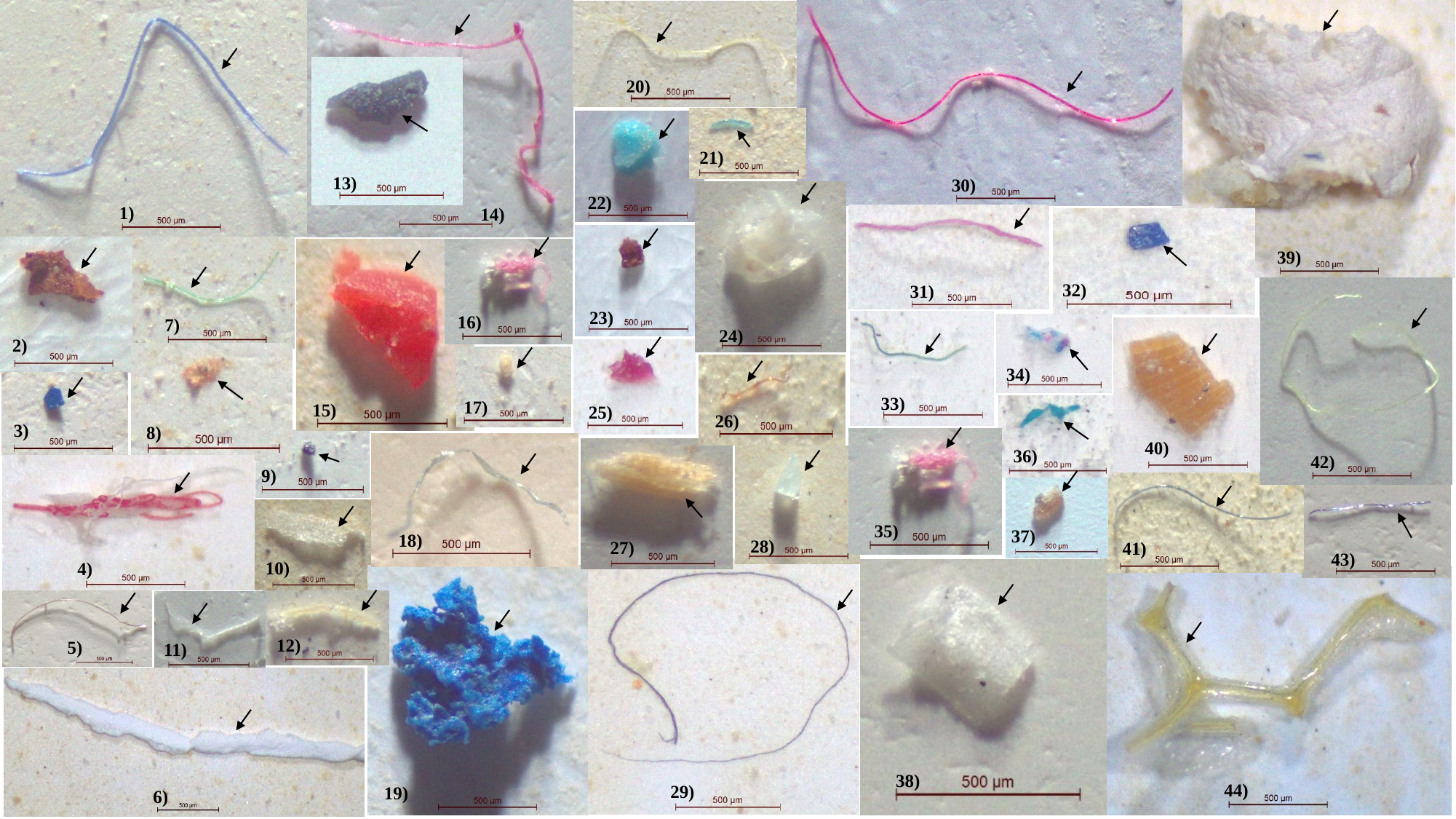

20)
21)
13)
30)
22)
1)
14)
39)
32)
31)
23)
16)
7)
24)
2)
34)
33)
17)
15)
25)
26)
3)
8)
40)
36)
42)
9)
35)
37)
18)
28)
27)
41)
43)
10)
4)
12)
5)
11)
38)
44)
29)
19)
6)

Supplement: Supplemental Information 2 [file peerj-10-12916-s002.pptx]
